# Who do patients depend on as they age and for what medical needs? An exploratory study of Chinese and Latino primary care patients

**DOI:** 10.1186/s12875-024-02411-7

**Published:** 2024-06-08

**Authors:** Jennifer Livaudais-Toman, Celia P. Kaplan, Leah S. Karliner

**Affiliations:** 1 Department of Medicine, Division of General Internal Medicine, University of California, San Francisco (UCSF), San Francisco, CA USA; 2 Multi-Ethnic Health Equity Research Center, UCSF, San Francisco, CA USA; 3 Helen Diller Family Comprehensive Cancer Center, UCSF, San Francisco, CA USA

**Keywords:** Aging, Caregiver roles, Informal caregivers, Limited English proficiency (LEP)

## Abstract

**Background:**

As the U.S. population ages, family members increasingly act as informal caregivers, particularly for minority patients and those with limited English proficiency (LEP). However, physicians often do not identify or engage caregivers until there is a health crisis. This study aims to further our understanding of characteristics associated with having a caregiver present at a primary care visit, and better understand the specific roles family caregivers engage in to support older Chinese and Latino primary care patients.

**Methods:**

Primary care patients were surveyed by telephone in a study of language access and communication. Participants included Chinese and Latino primary care patients (≥ 65 years old) from an academic general medicine practice. We asked patients if anyone was in the room with them during their most recent primary care visit (yes = caregiver accompanied). We asked about caregiving support for various needs, and examined associations of patient and visit characteristics with being accompanied, and frequency of caregiver support roles overall and by caregiver accompaniment.

**Results:**

Among 906 participants, 80% preferred a non-English language, 64% were women, 88% had Medicare, and mean age was 76 years (range 65–97). 43% were accompanied to their most recent visit. Speaking English ‘not at all’ vs. ‘very well’ was associated with being caregiver accompanied (OR 3.5; 95% CI 1.3–9.7), as was older age ≥ 75 vs. 65–74 (OR 2.7; 95% CI 2.0-3.7). The most common roles being supported by caregivers included: transportation to medical appointments (63%), helping with medical decisions (60%), and talking with the doctor about the patient’s medical care (54%). Even among unaccompanied patients, substantial proportions reported caregiver support with medical decisions (45%), talking with the doctor (33%), and medical needs at home (26%).

**Conclusions:**

Opportunities for physicians to engage caregivers who have active support roles may be missed, especially if those caregivers are not present at the visit. Future interventions should aim to help physicians identify which patients have caregivers and for what needs, so they may effectively engage caregivers before a health crisis occurs.

## Background

More than half of U.S. adults over the age of 65 need help with at least one daily activity [1]. As the U.S. population ages, family members are becoming increasingly involved in caregiving functions, particularly for racial/ethnic minority patients and those with limited English proficiency (LEP) [2–4]. Although many professional societies have advocated for including and supporting caregivers in healthcare [5], most health systems do not systematically integrate identification of, communication with, or support for caregivers into their approach to patient care. Further, despite the importance of family-centered decision making for older patients from Chinese and Latino cultures [4, 6], family caregivers are not often formally involved in care plans and communication for these patients until there is a health crisis [7]. 

Prior studies on patients and their caregivers have been limited to specific serious illnesses, including cancer, dementia, or stroke [8–10], or have not focused on racially or ethnically diverse LEP populations in primary care [11]. Notably, the largest number of older adults requiring family caregiver assistance with at least one task have neither diagnosed dementia nor disability [12]. 

In addition, 67 million Americans speak a language other than English at home [13] and 38% of these individuals are considered to have limited English proficiency (LEP) [13]. Patients with LEP may be more likely to bring family members or friends to their appointments, to help with interpretation and to navigate the complexities of the healthcare system [3, 4]. Yet, the family accompanying patients with LEP to their visits, may not be the same as family playing other support roles for patients at home [4]. 

As primary care is often the initial point of healthcare contact for older adults and their families, it is important to identify primary care patients who have caregivers and caregiving needs, so that physicians can engage those caregivers as part of the patient’s care team [7]. Complicating matters, physicians may have concerns about patient autonomy and privacy in considering broaching the topic with patients and engaging caregivers [5, 14]. At the same time, they may be unaware of the important family members or friends in a patient’s life, which individuals are providing caregiving support to the patient, and what roles those caregivers play, limiting their care plans with patients.

To further our understanding of current caregiver support practices among diverse older adults, we leveraged data from ethnically Chinese and Latino primary care patients collected as part of a study of language access and communication in primary care. Our goal was to elucidate patient characteristics associated with having a caregiver present at a primary care visit among ethnically Chinese and Latino patients with LEP, and better understand the specific support roles being met by different types of caregivers for these patients. We also aimed to understand how support might differ for patients who are accompanied compared to those unaccompanied to their visit.

## Methods

### Study design and setting

We used an existing database of 1,475 primary care patient telephone interviews from the Language Access Systems Improvement (LASI) study, which has been described previously [15–17]. LASI was designed as a natural experiment to evaluate the impact in primary care of a system-based quality improvement initiative aimed at increasing appropriate utilization of clinician non-English language skills and of professional interpreters. This study was approved by the University of California San Francisco (UCSF) Institutional Review Board Committee on Human Subjects.

### Participants

The LASI study recruited English, Chinese or Spanish-speaking primary care patients from the UCSF General Internal Medicine (GIM) practice to participate in a post-visit (within 1 week after a primary care visit) structured telephone interview during two timeframes (January 2014-March 2014 (pre-LASI); February 2016-June 2017 (post-LASI)). Patients were included if they were 40 years of age or older, self-identified as Chinese or Latino; were primary care patients in the GIM study practice with a primary care clinician who had self-reported their own non-English language skills; had a working telephone number and current address in the electronic medical record (EMR). Patients were excluded if: their primary care clinician opted out of having either their language data or their patients included in the study; patient’s hearing was too impaired to participate in a telephone interview; patient was unable to cognitively follow and answer an interviewer’s questions on the telephone. For this analysis, participants younger than 65 years of age were also excluded. The final sample for this analysis included all ethnically Chinese and Latino primary care patients ≥ 65 years from both study timeframes, who completed telephone surveys within 1 week after their primary care visit.

### Data collection and study variables

Demographic characteristics were collected from participants via telephone interview and included: preferred language for healthcare (English, Spanish, Cantonese, Mandarin), how well patient spoke English (not at all, not well, well, very well), age, gender, and education (< high school, high school/GED, AA/some college, ≥college).

Visit and utilization characteristics were collected from patients’ EMR and included: whether patient saw their own primary care provider (PCP), primary health insurance (private, Medicare, Medi-Cal), number of comorbidities, and frequency of primary care visits in the last 12 months. PCP characteristics included faculty status (Attending/Nurse Practitioner (NP) or Resident) and gender of doctor seen.

The telephone survey was conducted in the participant’s preferred language by a bilingual-bicultural trained research assistant who called the participant within 1 week after the primary care appointment. The survey took 10–20 min to complete and asked the following question, with a close ended response: was anyone was in the room with you during your primary care visit? (yes or no). If the response was yes, we considered them to be caregiver accompanied. The telephone survey also asked about caregiving support for various roles, with close ended responses: who helps you make important medical decisions; who talks with your doctor about your medical care; who helps you take your medications; who helps you at home with your other health or medical needs; who helps you schedule medical appointments; who helps you with transportation to your medical appointments. Response options included: spouse/partner, adult child, paid caregiver, other (other relative, friend, or other not specified) or nobody helps.

### Statistical analysis

We used descriptive statistics to describe the sample, and examined associations of patient and visit characteristics with being caregiver accompanied using both bivariate analysis and multivariable logistic regression modeling. We described caregiver support roles and examined frequency of caregiver support and type of caregiver for each role. Finally, we stratified the sample according to whether the patient was caregiver accompanied (or unaccompanied) and examined caregiver support roles for each group separately. All analyses were conducted using Stata Version 16.1.

## Results

### Participant characteristics and accompaniment to visit

Out of 1,475 original LASI participants, 569 were < 65 years of age and excluded from our analysis. Our final sample included the 906 ethnically Chinese and Latino primary care patients ages ≥ 65 years. The majority (80%) had a non-English language preference (Cantonese *n* = 359; Mandarin *n* = 190; Spanish *n* = 177), most were women (64%), most had Medicare (88%), most visits were with a faculty PCP (71%), and three-quarters saw their own PCP at the visit. Mean age was 76 years (range 65–97).

43% (*n* = 391) reported being accompanied to their most recent primary care visit. (Table 1) Patients who preferred speaking English were the least likely to be accompanied. In contrast, patients who reported speaking English “not at all” were most likely to be accompanied, as were older patients and those with the least education (Table 1).

**Table 1:** Caregiver Accompaniment among LASI Patients by Patient Characteristics (*N* = 906)

	No caregiver accompanied patient to visit (*N* = 515; 56.8%) *N* (%)	Caregiver accompanied patient to visit (*N* = 391; 43.2%) *N* (%)	*p*-value*
**Preferred language**			**< 0.001**
English	135 (75.0)	45 (25.0)	
Spanish-speaking	101 (57.1)	76 (42.9)	
Cantonese-speaking	174 (48.5)	185 (51.5)	
Mandarin-speaking	105 (55.3)	85 (44.7)	
**How well does patient speak English?**			**< 0.001**
Not at all	111 (39.4)	171 (60.6)	
Not well	230 (59.1)	159 (40.9)	
Well	88 (72.1)	34 (27.9)	
Very well	85 (75.9)	27 (24.1)	
**Age**			**< 0.001**
65–74 years	293 (71.5)	117 (28.5)	
75 + years	222 (44.8)	274 (55.2)	
**Gender**			0.4
Female	335 (58.1)	242 (41.9)	
Male	180 (54.7)	149 (45.3)	
**Education**			**< 0.001**
Less than high school	191 (49.6)	194 (50.4)	
High school/GED	74 (54.4)	62 (45.6)	
AA or some college	68 (62.4)	41 (37.6)	
College or higher	170 (66.1)	87 (33.9)	
**Patient saw PCP**			0.8
No	130 (56.3)	101 (43.7)	
Yes	385 (57.0)	290 (43.0)	
**Insurance**			0.1
Private	43 (69.4)	19 (30.6)	
Medicare	441 (55.5)	353 (44.5)	
Medi-Cal	31 (62.0)	19 (38.0)	
**Comorbidity count** **Mean ± SE**	2.8 ± 0.08	3.2 ± 0.11	**0.001**
**Frequency of visits in past 12 months**	3.6 ± 0.13	3.8 ± 0.14	0.4
**MD Faculty Status**			0.5
Attending/NP	360 (56.2)	281 (43.8)	
Resident	155 (58.5)	110 (41.5)	
**MD Gender**			0.9
Female	311 (57.1)	234 (42.9)	
Male	204 (56.5)	157 (43.5)	

*P-values account for clustering of patients by physician

In multivariable analysis (Table 2), speaking English ‘not at all’ vs. ‘very well’ remained significantly associated with being caregiver accompanied (OR 3.5; 95% CI 1.3–9.7), as did older age ≥ 75 vs. 65–74 (OR 2.7; 95% CI 2.0-3.7) and each additional comorbidity (OR 1.10; 95% CI 1.01–1.19). Speaking English ‘not well’ or ‘well’, language preferences and education were not significantly associated.

**Table 2:** Multivariable Adjusted Logistic Regression of Caregiver Accompaniment among LASI Patients ≥ 65 Years of Age (*N* = 886)

	Caregiver accompanied patient to visit OR (95% CI)	*p*-value*
**Preferred language**		
English	Ref	
Spanish-speaking	1.18 (0.49, 2.80)	0.714
Cantonese-speaking	1.33 (0.56, 3.18)	0.516
Mandarin-speaking	1.25 (0.51, 3.05)	0.631
**How well does patient speak English?**		
Very well	Ref	
Well	1.11 (0.53, 2.30)	0.785
Not well	1.62 (0.60, 4.32)	0.340
Not at all	3.52 (1.28, 9.67)	**0.015**
**Age**		
65–74 years	Ref	
75 + years	2.71 (2.00, 3.69)	**< 0.001**
**Gender**		
Female	Ref	
Male	1.21 (0.89, 1.65)	0.221
**Education**		
Less than high school	Ref	
High school/GED	1.15 (0.75, 1.77)	0.523
AA or some college	1.03 (0.63, 1.69)	0.907
College or higher	0.76 (0.51, 1.14)	0.190
**Patient saw PCP**		
No	Ref	
Yes	1.14 (0.81, 1.61)	0.438
**Insurance**		
Private	Ref	
Medicare	1.13 (0.61, 2.10)	0.701
Medi-Cal	0.97 (0.41, 2.30)	0.945
**Comorbidity count** **(continuous)**	1.10 (1.01, 1.19)	**0.024**
**Frequency of visits in past 12 months (continuous)**	0.97 (0.92, 1.03)	0.388
**MD Faculty Status**		
Attending/NP	Ref	
Resident	0.83 (0.60, 1.16)	0.275
**MD Gender**		
Female	Ref	
Male	1.00 (0.74, 1.34)	0.983

### Caregiver support roles

**Fig. 1:**
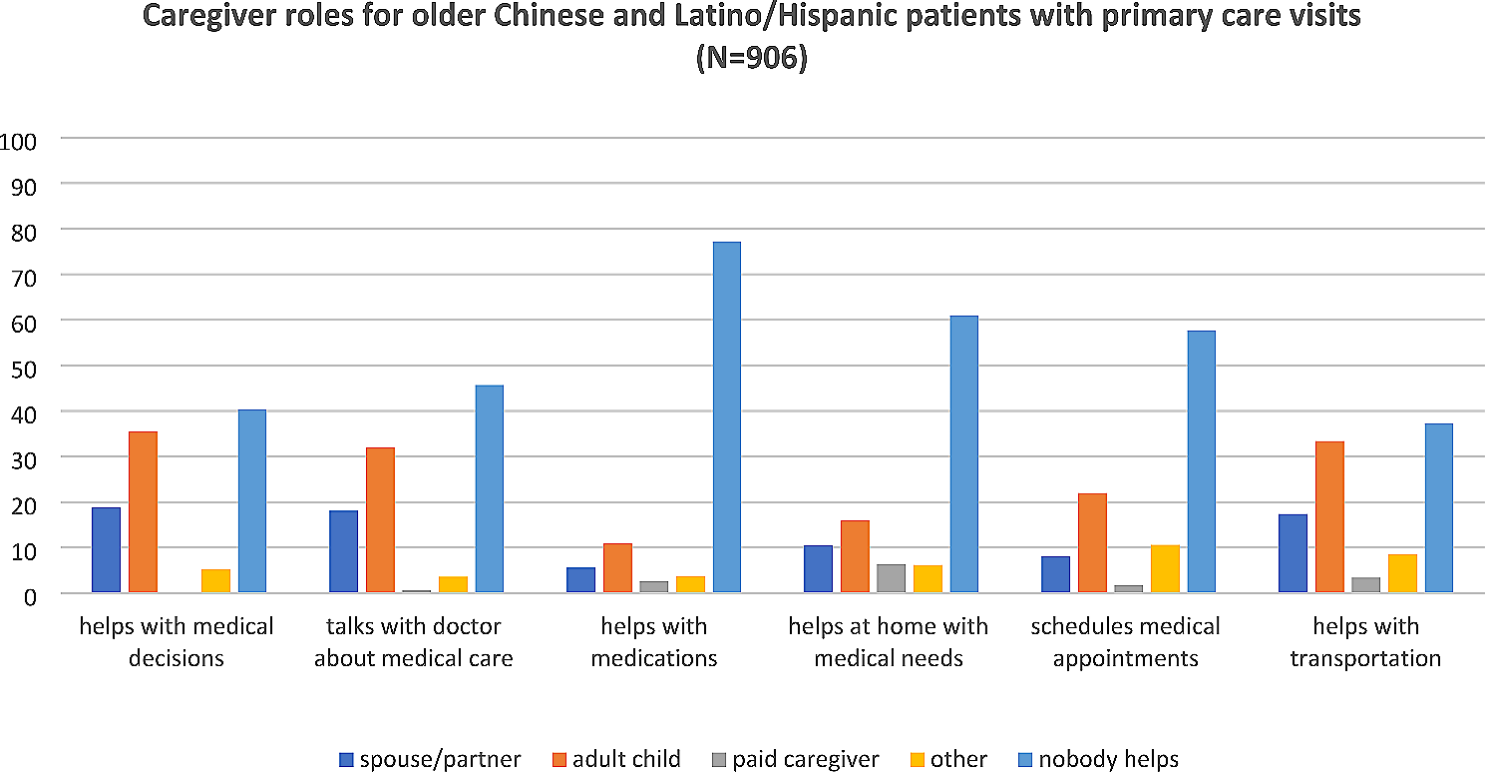
Caregiver roles for older Chinese and Latino patients with primary care visits

Overall, most patients (*n* = 741/ 82%) reported receiving caregiver support for at least one role. The most commonly reported caregiver support roles included helping with transportation to medical appointments (63%), helping with medical decisions (60%) and talking with doctors about the patient’s medical care (54%). Fewer participants reported that someone helped schedule medical appointments (42%), someone helped at home with other medical needs (39%), and someone helped them take medications at home (23%). For every caregiver support role, adult children followed by spouses/partners, most commonly met the need (Fig. 1).

**Fig. 2:**
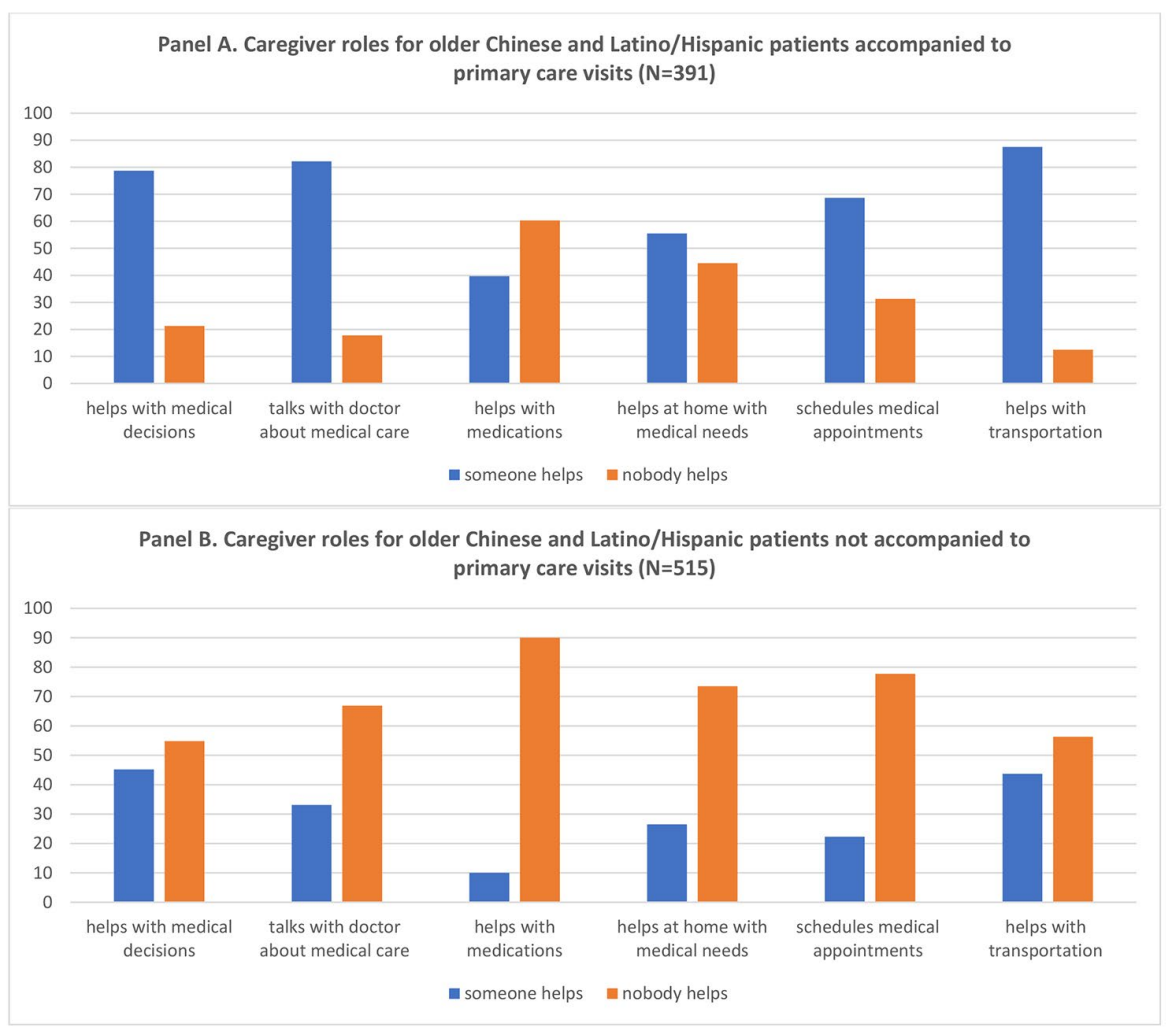
Panel **A**: Caregiver roles for older Chinese and Latino patients accompanied to primary care visits. Panel **B**: Caregiver roles for older Chinese and Latino patients not accompanied to primary care visits

Stratified by caregiver accompaniment, there were similar patterns of support roles for those who were and were not caregiver accompanied. While the percent reporting caregiver participation in support roles was highest for each role among those patients who were accompanied to their visit (Fig. 2: Panel A), even among patients not caregiver accompanied a substantial proportion reported caregiver support roles, with 45% reporting help with medical decisions, 33% help talking with doctors, and 26% help at home with medical needs (Fig. 2: Panel B).

## Discussion

In this population of older ethnically Chinese and Latino patients, almost half were accompanied to their primary care visits, and older, more medically complex patients were most likely to have a caregiver accompany them. These findings are consistent with prior research in other populations on the presence of family members during routine medical visits [18]. Notably, English proficiency was only associated with caregiver accompaniment for patients who reported not speaking English at all. Since the majority of visits took place after the LASI intervention, which successfully increased utilization of professional interpreters during visits [19], those with some English skills were likely accompanied to the visit for similar reasons as English speakers and not for language interpretation. Despite good access to interpreters during visits, those without any English skills may still have felt the need to have a caregiver with them to help navigate the healthcare system since LASI did not change use of interpreters outside of the clinical encounter [20]. 

Adult children appear to be taking on the majority of caregiving roles at home and interfacing with the health system, followed by spouses and partners. This is consistent with prior research characterizing family caregivers [2, 21]. According to a 2020 report on caregiving published by the American Association of Retired Persons, 50% of informal caregivers supported a parent or parent-in-law, while 12% supported a spouse or partner and 8% supported a grandparent or grandparent-in-law [2]. In addition to providing the majority of disability-related assistance to older adults, family caregivers also assist with health management activities, including arranging care, administering medications, and attending medical appointments [22]. These caregivers provide help because of a personal relationship with the patient rather than financial compensation [23]. 

In our study, being accompanied to the visit was also associated with engaging caregivers for other medical needs, including helping with medical decisions, talking with the doctor about medical care, helping with medications and other health needs at home, scheduling appointments and helping with transportation. As indicated, this is consistent with recent research suggesting that in addition to providing hands-on care, family caregivers are spending increasing amounts of time navigating, negotiating and managing healthcare services for patients [24, 25]. 

Notably, even patients who were not accompanied to their visits reported having caregivers helping them in some area of need, particularly with medical decisions and talking with their doctor about medical care. For these patients, opportunities for physicians to engage caregivers may be missed if the caregivers are not present at the visit. To our knowledge, the caregiving roles being met specifically for this subgroup of older Chinese and Latino patients who attend their medical visits alone have not been previously examined.

Because Chinese and Latino cultures tend to be more family-centric in making decisions [4, 6], it is important for primary care providers to integrate family caregivers into healthcare visits when they are present. When caregivers are not present, providers should inquire about care support needs and what roles caregivers might already fill for patients at home. This information will support PCPs in providing anticipatory guidance to patients and their caregivers as they age and require more assistance.

This study has several limitations. First, we only asked about who helps with various roles, not about care needs; thus, we do not know how many patients, particularly unaccompanied patients, have care support needs that are not being met. Notably, many patients who were accompanied to their visits (as well as those who were unaccompanied) indicated that no one helped them with certain medical tasks, including taking medications. Physicians should engage patients, and their caregivers when present, in discussions to determine challenges with medication adherence and the best way to overcome those challenges.

A second limitation of our study is that while we asked about a broad set of care roles, we may have missed elements of support. Third, LASI patients were cognitively intact enough to participate in a telephone interview so likely do not represent the growing population of older adults with more advanced memory and other cognitive impairments. Finally, because LASI was designed to focus on ethnically Chinese and Latino patients and took place in a single health system, we don’t know whether these results would hold true for patients from other groups or a broader geography.

## Conclusions

Despite these limitations, our results highlight the need to consider caregiving needs for all older Chinese and Latino and LEP patients in primary care, not only for those who are accompanied to their visits. Physicians and health systems should explore ways to identify patients’ caregivers earlier in care, understand their roles as well as patient preferences for future involvement in their care, and connect family caregivers to resources and support for their own health and wellbeing. Given the importance of family-centered decision making in Chinese and Latino cultures [4, 6], efforts should be made to help primary care teams support family centered decision making in culturally appropriate ways.

As a first step, physicians should engage those caregivers that do accompany their patients to medical appointments by assessing their ability and willingness to assist with potentially unmet needs at home. As a second step, physicians should not overlook potential caregiving needs for patients who attend their visits alone, and should ask patients about care needs and who, if anyone, helps them with those needs. Future efforts are necessary to develop reliable measures to assist primary care providers in understanding a patient’s family or social network for caregiving support, and to identify any roles that are not currently supported or not likely to be supported when needed, thus identifying a potential vulnerability before there is a health crisis.

## Data Availability

The datasets used and/or analyzed during the current study are available from the corresponding author on reasonable request.

## References

[1] Freedman VA, Spillman BC. Sep. Disability and care needs among older Americans. *Milbank Q*. 2014;92(3):509 – 41. 10.1111/1468-0009.12076. PMCID: PMC4221755. 10.1111/1468-0009.12076 PMC4221755 25199898

[2] *AARP and National Alliance for Caregiving: Caregiving in the United States 2020*. Accessed 2022. https://www.caregiving.org/research/caregiving-in-the-us/caregiving-in-the-us-2020/.

[3] Overcoming. *the challenges of providing care to limited English proficient patients*. 2021. *Quick Safety*. https://www.jointcommission.org/resources/news-and-multimedia/newsletters/newsletters/quick-safety/quick-safety--issue-13-overcoming-the-challenges-of-providing-care-to-lep-patients/overcoming-the-challenges-of-providing-care-to-lep-patients/#.YvQbaXbMI2x.

[4] Semere W, Napoles AM, Gregorich S, Livaudais-Toman J, Karliner L. Sep. Caregiving for Older Adults with Limited English Proficiency: Transitioning from Hospital to Home. *J Gen Intern Med*. 2019;34(9):1744–1750. 10.1007/s11606-019-05119-y. PMCID: PMC6712121. 10.1007/s11606-019-05119-y PMC6712121 31236893

[5] Mitnick S, Leffler C, Hood VL, American College of Physicians, Ethics P, Human Rights C. Mar. Family caregivers, patients and physicians: ethical guidance to optimize relationships. *J Gen Intern Med*. 2010;25(3):255 – 60. 10.1007/s11606-009-1206-3. PMCID: PMC2839338. 10.1007/s11606-009-1206-3 PMC2839338 20063128

[6] Pharr JR, Francis CD, Terry C, Clark MC. Culture, Caregiving, and Health: Exploring the Influence of Culture on Family Caregiver Experiences. *International Scholarly Research Notices*. 2014;2014.

[7] Schulz R, Eden J. Family Caregivers’ Interactions with Health Care and Long-Term Services and Supports. Families caring for an Aging America. National Academies; 2016. 27905704

[8] Gillespie R, Mullan J, Harrison L (2014). Managing medications: the role of informal caregivers of older adults and people living with dementia. A review of the literature. J Clin Nurs Dec.

[9] Kent EE, Rowland JH, Northouse L et al. Caring for caregivers and patients: Research and clinical priorities for informal cancer caregiving. *Cancer*. Jul 1. 2016;122(13):1987-95. 10.1002/cncr.29939. PMCID: PMC5597246. 10.1002/cncr.29939 PMC5597246 26991807

[10] Pindus DM, Mullis R, Lim L et al. Stroke survivors’ and informal caregivers’ experiences of primary care and community healthcare services - A systematic review and meta-ethnography. *PLoS One*. 2018;13(2):e0192533. 10.1371/journal.pone.0192533. PMCID: PMC5821463. 10.1371/journal.pone.0192533 PMC5821463 29466383

[11] Young HM, Bell JF, Whitney RL, Ridberg RA, Reed SC, Vitaliano PP. Social Determinants of Health: Underreported Heterogeneity in Systematic Reviews of Caregiver Interventions. *Gerontologist*. Feb 14. 2020;60(Suppl 1):S14-S28. 10.1093/geront/gnz148. PMCID: PMC7019663. 10.1093/geront/gnz148 PMC7019663 32057083

[12] Riffin C, Van Ness PH, Wolff JL, Fried T. Aug. Family and Other Unpaid Caregivers and Older Adults with and without Dementia and Disability. *J Am Geriatr Soc*. 2017;65(8):1821–1828. 10.1111/jgs.14910. PMCID: PMC5555780. 10.1111/jgs.14910 PMC5555780 28426910

[13] Zeigler K, Camarota SA. *67.3 Million in the United States Spoke a Foreign Language at Home in* 2018. 2019. https://cis.org/Report/673-Million-United-States-Spoke-Foreign-Language-Home-2018#:~:text=In%202018%2C%20a%20record%2067.3,and%20almost%20tripled%20since%201980.

[14] Parmar J, Anderson S, Abbasi M et al. Family Physician’s and Primary Care Team’s Perspectives on Supporting Family Caregivers in Primary Care Networks. *Int J Environ Res Public Health*. Mar 23. 2021;18(6)10.3390/ijerph18063293. PMCID: PMC8005195. 10.3390/ijerph18063293 PMC8005195 33806725

[15] Garcia ME, Hinton L, Gregorich SE, Livaudais-Toman J, Kaplan C, Karliner L. Apr. Unmet Mental Health Need Among Chinese and Latino Primary Care Patients: Intersection of Ethnicity, Gender, and English Proficiency. *J Gen Intern Med*. 2020;35(4):1245–1251. 10.1007/s11606-019-05483-9. PMCID: PMC7174511. 10.1007/s11606-019-05483-9 PMC7174511 31667737

[16] Nouri SS, Pathak S, Livaudais-Toman J et al. Use and Usefulness of After-Visit Summaries by Language and Health Literacy among Latinx and Chinese Primary Care Patients. *J Health Commun*. Aug 2. 2020;25(8):632–639. >. 2020;35(4):1245–1251. 10.1080/10810730.2020.1833385. PMCID: PMC8362332. 10.1080/10810730.2020.1833385 PMC8362332 33059522

[17] Pathak S, Gregorich SE, Diamond LC et al. Aug. Patient Perspectives on the Quality of Professional Interpretation: Results from LASI Study. *J Gen Intern Med*. 2021;36(8):2386–2391. 10.1007/s11606-020-06491-w. PMCID: PMC7845580. 10.1007/s11606-020-06491-w PMC7845580 33515189

[18] Wolff JL, Roter DL. Mar. Family presence in routine medical visits: a meta-analytical review. *Soc Sci Med*. 2011;72(6):823 – 31. 10.1016/j.socscimed.2011.01.015. PMCID: PMC3070824. 10.1016/j.socscimed.2011.01.015 PMC3070824 21353358

[19] Karliner L, Diamond L, Toman J (2021). Testing a program to improve patient-clinician communication for patients who speak Limited English. Patient-Centered Outcomes Res Inst (PCORI).

[20] Kornbluth L, Kaplan CP, Diamond L, Karliner LS. Jan. Communication methods between outpatients with limited-English proficiency and ancillary staff: LASI study results. *Patient Educ Couns*. 2022;105(1):246–249. 10.1016/j.pec.2021.05.001. PMCID: PMC8868014. 10.1016/j.pec.2021.05.001 PMC8868014 34023171

[21] Caregiver Statistics. : *Demographics*. 2016. https://www.caregiver.org/resource/caregiver-statistics-demographics/.

[22] Riffin C, Wolff JL, Pillemer KA. Feb. Assessing and Addressing Family Caregivers’ Needs and Risks in Primary Care. *J Am Geriatr Soc*. 2021;69(2):432–440. 10.1111/jgs.16945. PMCID: PMC8062767. 10.1111/jgs.16945 PMC8062767 33217776

[23] Wolff JL, Feder J, Schulz R (2016). Supporting family caregivers of older americans. N Engl J Med Dec.

[24] Schulz R, Beach SR, Friedman EM, Martsolf GR, Rodakowski J, James AE. 3rd. Changing Structures and Processes to Support Family Caregivers of Seriously Ill Patients. *J Palliat Med*. Mar 2018;21(S2):S36-S42. 10.1089/jpm.2017.0437. PMCID: PMC5756457. 10.1089/jpm.2017.0437 PMC5756457 29091533

[25] Taylor MG, Quesnel-Vallee A (2017). The Structural Burden of Caregiving: Shared challenges in the United States and Canada. Gerontologist Feb.

